# Overlapped Bayesian spatio-temporal models to detect crime spots and their possible risk factors based on the Opole Province, Poland, in the years 2015–2019

**DOI:** 10.1186/s40163-023-00189-0

**Published:** 2023-05-22

**Authors:** Rafał Drozdowski, Rafał Wielki, Andrzej Tukiendorf

**Affiliations:** 1 Opole Police Department, 1 Powolnego Street, 45-078 Opole, Poland; 2 Faculty of Law and Administration, University of Opole, 87a Katowicka Street, 45-060 Opole, Poland; 3 Institute of Health Sciences, Opole University, 68 Katowicka Street, 45-060 Opole, Poland

**Keywords:** Crime statistics, Crime mapping, Cold/hot-spot detection, Bayesian modeling

## Abstract

Geostatistical methods currently used in modern epidemiology were adopted in crime science using the example of the Opole province, Poland, in the years 2015–2019. In our research, we applied the Bayesian spatio-temporal random effects models to detect ‘cold-spots’ and ‘hot-spots’ of the recorded crime numbers (all categories), and to ascertain possible risk factors based on the available statistical population (demographic), socio-economic and infrastructure area characteristics. Overlapping two popular geostatistical models in the analysis, ‘cold-spot’ and ‘hot-spot’ administrative units were detected which displayed extreme differences in crime and growth rates over time. Additionally, using Bayesian modeling four categories of possible risk factors were identified in Opole. The established risk factors were the presence of doctors/medical personnel, road infrastructure, numbers of vehicles, and local migration. The analysis is directed toward both academic and police personnel as a proposal for an additional geostatistical control instrument supporting the management and deployment of local police based on easily available police crime records and public statistics.

**Supplementary Information:**

The online version contains supplementary material available at 10.1186/s40163-023-00189-0.

## Introduction

Mapping is a principal function in crime analysis. As reviewed by Wang (2012), the first choropleth maps of crimes (crimes against persons, crimes against property, and school instruction) were already being published in France in the 1820s. Since their inception, these maps were characterized by the nature of the research data, taking into account the ecological perspectives of crime, urban infrastructure, socioeconomic indicators, educational levels, poverty, transportation, routes, and ethnic and cultural variations. Weather and other contextual variables were also included. The prototypical mapping of crime-related variables, now widely used by urban ecologists, was created at university centers such as the University of Chicago (1915), while the first computer applications for mapping the distribution of crimes (in St. Louis, Missouri) was developed at Harvard University in 1967 (Clarke, 1999). Since that time, computerized crime mapping has experienced a huge boom in hardware investment and programming efforts, and present day police work would not be possible without these technologies.

When studying the statistical and geostatistical methods currently applied in criminology, one gains the impression that they do not differ from those used in modern epidemiology. For example, Moran’s *I* spatial autocorrelation index (Baller et al., 2001), distance statistics such as nearest neighbor analysis and Ripley’s *K* statistic (Levine, 2008), taxonomic approaches (Ratcliffe, 2004), *K*-means clustering (Levine, 2008), temporal and spatial hotspots (Almanie et al., 2015; Braga & Weisburd, 2020; Brantingham & Brantingham, 1981; Chainey et al., 2008; Eck et al., 2005; Ratcliffe & McCullagh, 1998; Sherman et al., 1989), spatial regression modeling (Baller et al., 2001; Mburu & Helbich, 2016), change-point models (Cork, 1999), and Bayesian spatio-temporal regressions (Hu et al., 2018; Law et al., 2013) are all typically used in the epidemiology of chronic diseases. Moreover, some reports directly adopt measures of health studies, for example, Malczewski and Poetz (2005) on the socio-economic factors and residential burglaries, while SatScan software (Kuldorff, 1997) is recommended for use in parallel in both fields of science (Eck et al., 2005). Other authors also confirm the usefulness of using epidemiological models in criminological research (Hu et al., 2018; Law et al., 2013).

Crime mapping in Poland also has a quite long, but not rich history. Indeed, the first Polish articles of international scope only date back to the 1980s (Bartnicki, 1986). The most important criminal geostatistics made available abroad include those listed in the Atlas of Crime in Poland (Siemaszko et al., 2015) which is periodically issued by the Institute of Justice in Warsaw. Furthermore, Sypion-Dutkowska and Leitner (2017) had work published that assessed the influence land use has on the spatial distribution of urban crime in the city of Szczecin, Poland. However, we agree with the opinion by (Mordwa, 2016) that “Polish geography of crime is poorly developed” and we want to fill this scientific niche with an exemplary geostatic analysis of general regional crime against a demographic and socio-economic background. The example we employ is based on the data from the years 2015–2019 of the Opole province, Poland.

Although newer and newer publications using different methodological approaches in quantitative criminology have appeared in the literature (see Duan et al., 2017, on artificial neural networks, or Yue et al., 2022, based on a negative binomial regression), the advantages of the Bayesian methods are still underestimated. Therefore, in the present research, we adopted the Bayesian spatio-temporal random effects model to detect ‘cold-spots’ and ‘hot-spots’ of recorded crime by overlapping relative risks (RRs) and growth rates (GRs). Additionally, we analyzed the available underlying demographic and socioeconomic conditions that might have stood (directly or indirectly) behind the possible differences in crime levels in the Opole region over time. Together, as for the first time that Bayesian modeling was successfully launched in quantitative criminology by Law et al. (2013), and afterwards by Hu et al. (2018), we believe that the adopted epidemiological approach will be applicable for analyzing local patterns of crime from a short time period, and, hence, bring significant benefits to policing analyses.

## Materials

The study region is the Opole province, about 9412 km^2^, located in the southwestern part of Poland. Opole presently consists of 12 administrative counties and 104 communities. It is populated by approximately 983 K inhabitants (2019). Opole is not recognized as one of the elite political, economic or educational centers of Poland, and its role in the national development is not leading. However, in recent years some key industrial investments were made in the region, especially in energy (a power plant construction), which has resulted in the employment of additional workers of various nationalities (a few thousand people).

Information on recorded crime in the region originates from the Opole Police Department. “Statistics Poland” was our data source for the demographic and socio-economic characteristics of the population and regional infrastructure. The basic recorded crime data consisted of incidents of crime (all categories) reported to the police for the 5 years spanning just before the COVID-19 pandemic from January 1, 2015 to December 31, 2019 (Table 1).

**Table 1:** Crime and population numbers in the Opole province, Poland, in the years 2015–2019 (p > 0.05)

Year	2015	2016	2017	2018	2019
Crimes	27,411	28,063	28,399	24,428	24,702
Population	996,011	993,036	990,069	986,506	982,626

All crime incidents were coded by year of offense and accompanied by a street address that was geocoded to a particular administrative unit (community) and then analyzed in the context of the crime risk and its increment.

This study also considered 64 available contextual characteristics (demographic, living, business, cultural, budget, employment, infrastructural, automotive, touristic, real estate, and migration) as the potential explanatory (continuous and count) variables within the same quinquennium 2015–2019 (Table 2).

**Table 2:** Characteristics and specification of explanatory (continuous and count) variables

Characteristics	# of expl. var	Specification
Demographics	6	population density per 1 km^2^; marriages/divorces/deaths per 1 K/% of population of working age/ # of females
Living	12	# of flats per 1 K/per 1 K contracted marriages; average flat area/average of rooms in 1 flat; new residential buildings/new flat area/new flat area per 1 K
Business	10	all registered entities/per 1 K/per 1 K in working age and removed entities; newly registered entities per 1 K; natural persons in business per 1 K/per 1 K of working age; newly/all registered entities/in agri-food sector/employing up to 9 persons
Cultural	6	population per 1 library; book collection/readers of libraries per 1 K; volume of book borrowing for 1 reader; # of population engaged in public events per 1 K/per cultural and educational institution
Budget	2	budget expenditure/revenues
Employment	4	doctors/medical personnel per 10 K; doctors/medical personnel working at basic workplace per 10 K
Infrastructural	7	roads with hard surface/improved surface/per 100 km^2^/per 10 K; roads with hard surface/improved surface/unsurfaced in km
Automotive	4	# of motorcycles/passenger cars/trucks/tractors
Touristic	1	average daily hotel rate
Real estate	6	average for 1 m^2^/price/total price of residential premises sold on the primary/secondary market
Migration	6	total registration/deregistration in pre-working/working/retirement age
Total:	64	explanatory variables

The choice of these variables was based on a review of the relevant literature and available data in Statistics Poland. The derived data were intentionally used to reflect the variation in the risk of regional crime.

In order to avoid an overabundance of research results, and to accentuate the methods of statistical analysis, this study did not break down crime data into sub-types and sub-periods, as this would significantly expand the framework of this paper.

## Methods

The analysis of geographical variations in rates of crime is useful in the formulation of causal hypotheses. Crime mapping aims to elucidate the geographical distribution of underlying crime rates and to identify areas with low or high rates.

The main conventional approach is maps of standardized rates based on Poisson inference. This method has the advantage of providing estimates of the parameters of interest, but raises a problem. First, for rare events and for small areas, variation in the observed number of events exceeds the expected Poisson inference. Because the conventional standard ratio of the outcome of interest may in part be distorted by small denominator populations, in a given area, variation in the observed numbers is due partly to Poisson sampling, but also to extra-Poisson variation (Congdon, 2000; Mollié, 1999).

To overcome high chance variations in frequentist methods, Bayesian approaches have also been proposed in crime mapping (Hu et al., 2018; Law et al., 2013). They consist of considering, in addition to the observed events in each area, prior information in the variability of crime rates in the overall map (classical approaches model dependence among observations as part of the single-stage likelihood, while Bayesian approaches typically assume observations to be conditionally independent given the model parameters, and subsequently incorporate dependence at the second stage as part of the prior distribution—see Carlin & Louis, 2000, for example). What is more, Bayesian area-specific estimates integrate the two types of information. They are close to the standardized rates when based upon a large number of events. However, with few events prior information on the overall map will dominate, thereby shrinking standardized rates towards the overall mean rate (Clayton & Kaldor, 1987). Consequently, Bayesian models are stabilizing (smoothing) risk maps and producing robust and reliable estimation of the outcomes of interest in a small area, even when based on small sample sizes (Ancelet et al., 2012; Kang et al., 2016). Compared to the frequentist approach, the use of prior distributions helps strengthen inferences about the true value of the parameter and ensures that all relevant information is included (Gurrin et al., 2000; Waller & Carlin, 2010). Moreover, the frequentist maximum likelihood estimate over space or space–time treats each area or area-time as a separate and isolated entity. The resulting estimate of relative risk takes no account of the average for the region in which it is located or of associations with risks in neighboring areas or area-times. However, in the event of spatially structured risks (crime outcomes may exhibit spatial correlation), relative risks for spatially adjacent areas may be assumed dependent (the log relative risks are entirely due to spatially correlated effects—see Congdon, 2000, for details).

However, as experts point out, in spatial regression analysis, frequentist models such as spatial lag or spatial error model have limitations because the response variable needs to be continuous (i.e. rate, not count) and should follow a normal distribution (Law et al., 2013). Moreover, they are designed for processing observations from one time period only and they treat parameters (regression coefficients) as fixed values, which contrasts the unknown and unfixed quantities that occur in reality. Therefore, models fail to analyze available information systematically and are restrictive for computations (the models have to be tractable for solving the equation—see Carlin & Louis, 2000).

In contrast to the frequentist approach, Bayesian methods treat data as fixed and unknown quantities or parameters as random variables expressed in terms of probabilities and can conveniently process observations from more than one location and time period (by assuming a hierarchical structure, they allow integration of available information and analyses into a single model including purely spatial, purely temporal, and spatio-temporal interaction). The other reason why Bayesian methods should be used in crime research is the fact that they analyze continuous outcome variables (such as crime rates) as well as discrete outcome variables that follow Poisson, Bernoulli, or binomial distributions (they are preferred over analyzing ‘‘calculated’’ rates because rates depend heavily on the denominator used—see Law et al., 2013).

The above facts provide evidence that there are many reasons why Bayesian modelling is a useful framework for risk mapping. One of the most popular forms for expressing spatial dependence is via an intrinsic conditional autoregressive (CAR) model specified in terms of differences in risk between a pair of the two units of the neighboring areas and modelled conditionally as a spatial random variable. When areal data has a spatial structure such as observations from neighboring regions, they exhibit a higher correlation than distant regions (Besag, 1974).

Recognizing the similarity to epidemiological diseases, we consider spatio-temporal Bayesian modeling (i.e. models of spatial outcomes involving the evolution over time of such outcomes) following Bailey (2008), also of great practical and potential value in the risk analysis of crime. In the simplest temporal extension of the purely spatial Bayesian disease mapping model, a temporally unstructured time effect into the model is included. Given crime counts *y*_*it*_ (in areas *i* and years *t*) and corresponding expected numbers of cases *e*_*it*_ (derived from suitable reference populations) the model is:

$$y_{it} \sim {\text{Poisson}}(\mu_{it} ) = {\text{Poisson}}\;(e_{it} \rho_{it} ),$$

$${\text{log}}(\mu_{it} ) \, = {\text{ log}}\left( {e_{it} } \right) \, + {\text{ log}}(\rho_{it} ) \, = {\text{ log}}\left( {e_{it} } \right) \, + \alpha + \phi_{i} + \nu_{i} + \delta_{t} ,$$

$$\alpha \sim {\text{Uniform}}\;( - \infty , \, + \infty ),$$

$$\phi_{i} \sim {\text{ Normal}}\;(0,\;\sigma_{\phi }^{2} ),$$

$$\nu_{i} \sim {\text{ CAR}}(0,\;\sigma_{\nu}^{2} ),$$

$$\delta_{t} \sim {\text{ Normal}}\;(0,\sigma_{\delta }^{{2}} ),{\text{ for } t} = { 2}, \, \ldots ,{\,T \text { with}}\;\delta_{{1}} = 0\;\left( {\text{as a baseline}} \right),$$

so relative risks are *ρ*_*it*_ = exp(*α* + *ϕ*_*i*_ + *ν*_*i*_ + *δ*_*t*_) with *ρ*_*i*1_ = exp(*α* + *ϕ*_*i*_ + *ν*_*i*_), where *α* is the mean log relative risk over all areas, *ϕ*_*i*_—a zero mean spatially unstructured (or exchangeable) log relative risk of area *i* compared to the map as a whole with variance *σ*_*ϕ*_, *ν*_*i*_—corresponding spatially structured (or non-exchangeable) random effect with variance *σ*_*ν*_ (controlling the strength of local spatial dependence), and *δ*_*t*_ is a zero mean temporally unstructured time effect in time period *t* > 1 with variance *σ*_*δ*_.

Where interest focuses on modelling trends in the relative risk in relation to the reference levels, then a stronger parametric structure on the temporal effects can be imposed. In modelling time trends in the outcome variable, which may be different between areas, Congdon (2003) has specified an area specific random growth rate. Particularly, in this approach, a linear trend, uniform across all areas is included in the model

$${\text{log}}(\mu_{it} ) \, = {\text{ log}}\left( {e_{it} } \right) \, + \alpha + \phi_{i} + \gamma t,$$

with *γ* ~ Normal(0, *σ*_*γ*_^2^) and all other priors as earlier, was differentiated between areas

$${\text{log}}(\mu_{it} ) \, = {\text{ log}}\left( {e_{it} } \right) \, + \alpha + \phi_{i} + \gamma_{i} t,$$

with exchangeable priors *γ*_*i*_ ~ Normal(*μ*_*γ*_, *σ*_*γ*_^2^), where *μ*_*γ*_ is the overall average growth rate.

However, if time trends are expected to be differentiated in a spatially distinct pattern, then the *γ*_*i*_ might be assumed to be spatially dependent, for example with intrinsic CAR (Congdon, 2003).

In turn, to study the possible risk factors of the analyzed crime variation we used a simple extension of the crime mapping model discussed earlier to include a particular covariate *x*_*it*_ measured in each area *i* and time period *t* (year of observation) with

$${\text{log}}(\mu_{it} ) \, = {\text{ log}}\left( {e_{it} } \right) \, + \alpha + \beta x_{it} + \phi_{i} + \nu_{i} + \delta_{t} ,$$

where *β* represents the relationship (slope) with *x*_*it*_ over the region and time period. In our work the relative risks and growth rates were plotted graphically in choropleth thematic maps.

All computations were conducted with WinBUGS software (Spiegelhalter et al., 2003) via a Markov Chain Monte Carlo (MCMC) simulation, which allows algorithms to generate dependent samples from the posterior distribution of the models. Due to its highly parameterized modelling, it was run for “burn in” for 1000 iterations and the subsequent 10,000 “production run” samples were utilized in our research. Herein, the performance of the MCMC simulation of the Gibbs sampler was diagnosed with the Gelman-Rubin statistic available within the WinBUGS software. Additionally, each chain was tested for one-sided p-value by employing the internal ‘step’ function to establish the significance of the particular covariate’s effect (slope regression parameter). Due to the requirement to perform a large number of statistical tests, to reduce the possibility that some might have p < 0.05 purely by chance, Bonferroni correction was applied in the analysis.

## Results

**Fig. 1:**
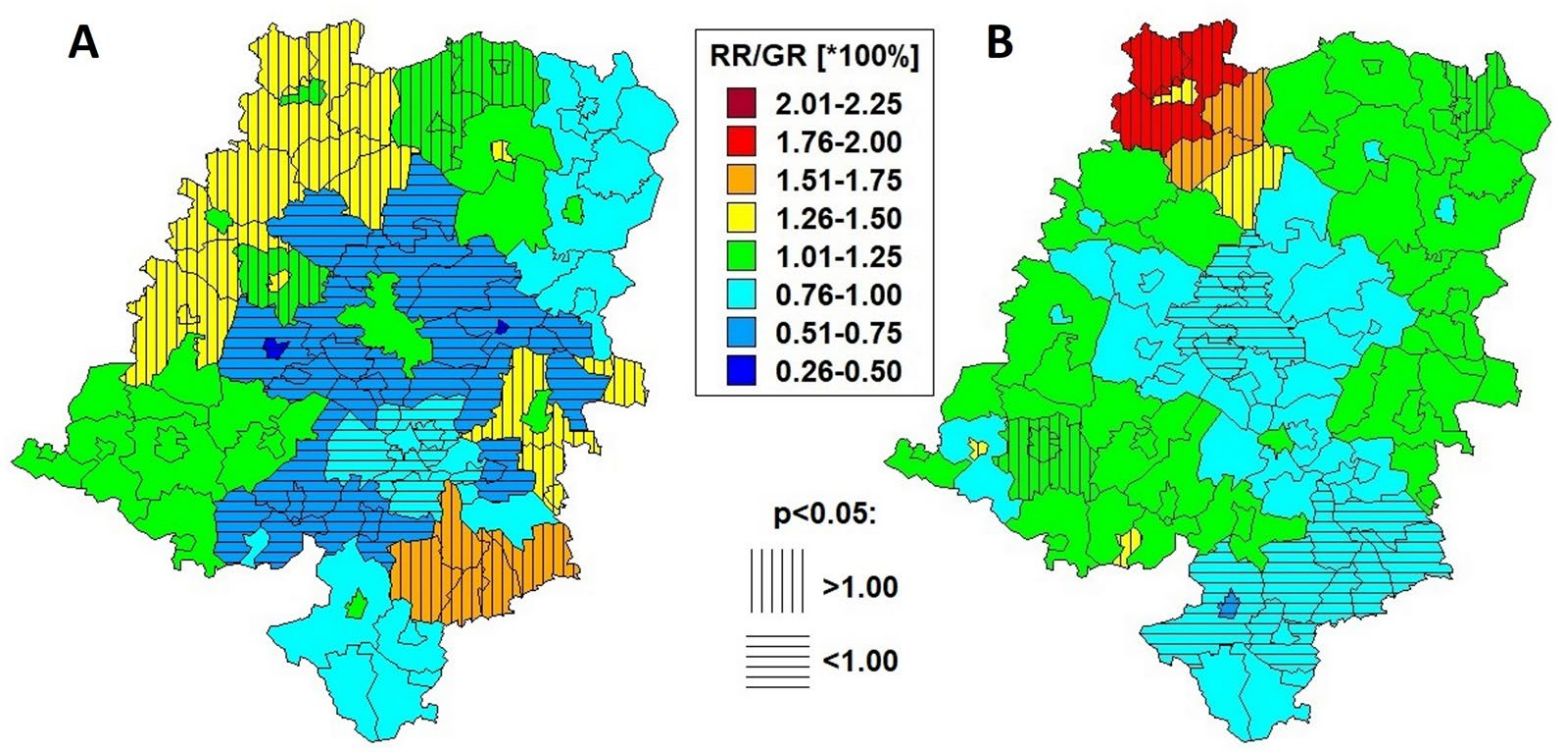
Spatio-temporal models of relative risks (**A**) and growth rates (**B**) of total crime in Opole, Poland, from 2015 to 2019. The maps are thematically represented by the arbitrary equidistant ranges of crime risk levels to show their spatial risk diversity (left map) and changes over times (right map), respectively. If the RR or GR of observed/expected value is significantly smaller (p < 0.05) than 1.0 = 100% (horizontally hatched units), then there is said to be a “moderate risk”, whereas if RR/GR > 1.0 (vertically hatched areas), then we are dealing with an “excessed risk” of crime. The overlapping of both of the same type of hatched surfaces reveals cold and hot-spots of space and time crime risks documented in the next thematic maps in Fig. 2

Estimates of the geostatistical models of relative risks and growth rates in the form of local patterns of total crime at the community level for Opole, Poland, over the time period 2015–2019 were established (Fig. 1A, B, respectively).

**Fig. 2:**
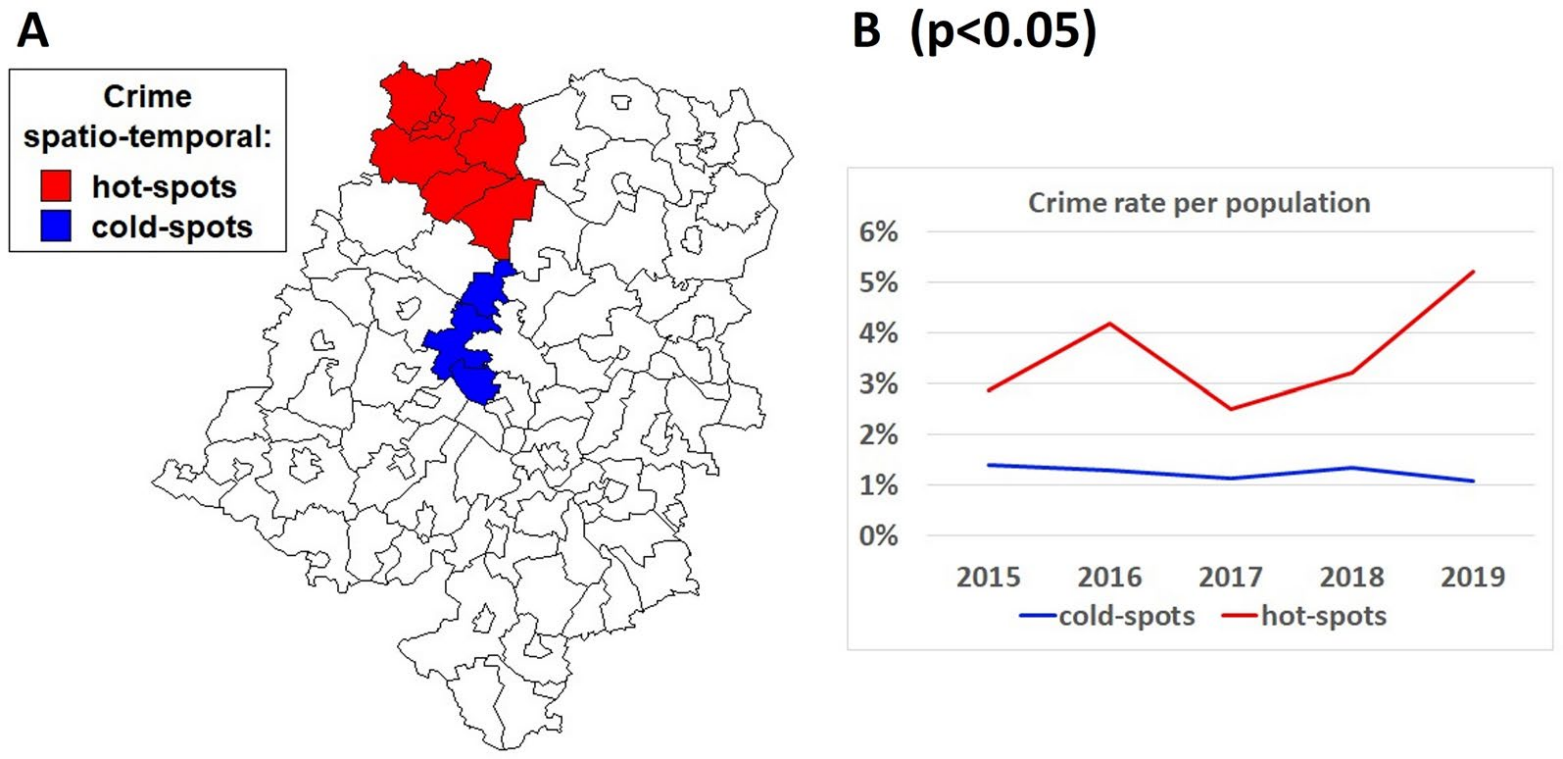
Cold-spots and hot-spots of total crime in Opole, Poland, in the years 2015–2019. The map in **A** is the result of the overlap of the same vertical or horizontal hatched surfaces representing territories with significantly moderate (p < 0.05) or excessive crime risks in space and over time, respectively (documented in the previous thematic maps in Fig. 1). In turn, in **B**, the most significant difference in crime rates per reference population is shown between cold and hot-spot areas found in the combined spatio-temporal Bayesian approach

By overlapping both RR and GR maps (Fig. 1A, B), on the basis of statistically significant estimates (p < 0.05), we were able to define the most ‘cold-spot’ (Dąbrowa, Dobrzeń Wielki, and Komprachcice) and ‘hot-spot’ (Domaszowice, Pokój, Świerczów, Namysłów city, Namysłów village, and Wilków) communities with regard to current and projected crime increases in Opole for the years 2015–2019 (Fig. 2A). The evident differences in total crime rates between cold-spots and hot-spots are additionally presented in Fig. 2B.

After Bonferroni correction, based on the available dataset (see the list in Table 2), the following statistically significant (p < 0.05) spatio-temporal regression coefficient (*β*) of risk factors were found to explain RRs of total crime in Opole between 2015 and 2019 (Table 3).

**Table 3:** Statistically significant (p < 0.05) regression coefficient (*β*) with 95% credible intervals (95%CIs) of risk factors for total crime in Opole between 2015 and 2019 (after Bonferroni correction)

Risk factor	Regr. coeff. (*β*)	95%CI
Doctors at basic workplace per 10 K	0.015	(0.008, 0.021)
Doctors (total) per 10 K	0.014	(0.010, 0.018)
Medical personnel at basic workplace per 10 K	0.006	(0.003, 0.009)
Roads with hard surface [km]	− 0.0010	(− 0.0015, − 0.0004)
Roads with improved surface [km]	− 0.0010	(− 0.0015, − 0.0004)
Roads unsurfaced [km]	− 0.0117	(− 0.0164, − 0.0069)
Passenger cars [#]	− 0.006	(− 0.010, − 0.003)
Trucks [#]	− 0.043	(− 0.068, − 0.017)
Tractors [#]	− 0.148	(− 0.217, − 0.075)
Registration of pre-working age [#]	− 0.0022	(− 0.0030, − 0.0013)
Registration of working age [#]	− 0.0009	(− 0.0012, − 0.0006)
Deregistration of pre-working age [#]	− 0.0032	(− 0.0045, − 0.0019)
Deregistration of working age [#]	− 0.0008	(− 0.0013, − 0.0003)

Following the reported Bayesian estimates of the regression coefficients in Table 3, it can be seen that 13 out of the 64 analyzed risk factors could statistically influence changes in the crime rate in Opole in the years 2015–2019. Generally speaking, medical care indicators have stood behind a positive impact on the scale of crime in the province. The remaining risk factors—grouped as road infrastructure, the number of vehicles, and population migrations—hold a negative correlation with crimes recorded in terms of the space and time under study.

## Discussion

The Opole police’s crime information usually relies on simple statistical analyzes and measurements (descriptive statistics and graphics). For this reason, conclusions from such analyzes are not drawn on the assumptions of mathematical statistics (i.e. based on verification of hypotheses or estimation). Therefore, in order to carry out statistical inference using such data, proper scientific cooperation between the police and statisticians is needed (it could consist in the transfer of police data for advanced and more reliable statistical analyses).

Bayesian methods can significantly simplify police work in that their application can aid in precisely detecting areas with extreme levels of criminal offenses and identify their background or possible risk factors. The results of these studies can be helpful in the appropriate management or relocation of police personnel to improve work efficiency and crime detection. Moreover, they can be used in deeper cause and effect analyzes. However, as suggested by Grana and Windell (2007), scientific cooperation between the staff and professional statisticians and the use of modern computing techniques is necessary. Hence, it seems that such statistical operations should be carried out more routinely and not be the product of strong encouragement from one side or the other by the persons concerned. Of importance is that the obtained statistical results are reliable and are not based on undocumented premises that may mislead police commanders and the public.

In this study we have shown the fruits of the research of such a collaboration, and in using advanced, but well-known geostatistical methods, we have drawn -highly captivating academic and investigative conclusions for police work on crime in a region with a population of a million and few years of observation. In the context of the proposed computational technique, combining two popular geostatistical models revealing ‘cold-spot’ and ‘hot-spot’ geographical patterns seems to be particularly attractive in the conducted analysis, finally allowing for the display of extreme differences in crime and growth rates over time. Such geostatistical opportunities can be easily exploited in the other mentioned logistic operations to achieve a balance in ensuring public order in the region.

Referring to other results obtained, it is difficult for us to explain the positive correlation between the number of doctors and medical personnel and the level of crime in Opole. If we accept that a greater density of these specialists is related to the extensive infrastructure of medical facilities, then our results are in contradiction with Bondurant et al. (2018) reporting that “an increase in the number of treatment facilities causes a reduction in both violent and financially-motivated crime”. On the other hand, this result could herald some concern in the debate at police or military command levels or with the public about the fact that healthcare facilities are recognized as critical infrastructure and these institutions must be given special protection (Atlas, 2013). In view of this we can expect that the correlation found is not related to crimes committed by medical personnel, but has its origin in the medical infrastructure, which is exposed to a high risk of robberies or other criminal behavior.

What is more, the inverse relationship between the length of local roads and a lower crime rate is opposite to the geostatistical outcomes obtained by Davies and Johnson (2014), who suggested that more linear streets and hence a less dense road infrastructure and shorter total distances were generally found to generate a lower risk of victimization. Beavon et al. (1994) have also confirmed this observation, stating that “crime was higher in more accessible and highly used areas and lower in the less accessible and less used areas”, while Johnson and Bowers (2010) point out even more precisely that the risk of residential burglary is higher on major roads and roads that are immediately connected to them. Following these reports, the density of the public road network, hence, increases the risk of burglaries, while their connection to local roads of limited access reduces this risk. But in order not to distance our results too much we must also look at another aspect of roads as sinuous roads are considered safer than linear (Armitage, 2017), which must be longer and are typical for less populated or highland areas. Nevertheless, not everything can be compared because the cited observation refers to property crimes, while our result is about overall crime numbers. Unfortunately, the impact of road infrastructure on crime risk cannot either be directly linked with the economic factors, which are not statistical relationships in the performed Bayesian modelling. So our results require deeper investigation and some questions remain unanswered. In turn, the number of crimes decreases with the absolute number of vehicles registered in geographic units. 

Firstly, this may be related to the wealth of the residents (the more vehicles they have, the richer they are), but also it is a derivative of the previously considered correlation about the length of local roads on which vehicles travel (the number of vehicles must have had forced the expansion of road infrastructure in these units). Anyway, we find these results puzzling. One might have expected that the increase in the number of registered cars could have resulted in a greater number of motor vehicle incidents, vehicle thefts or car break-ins. Still, while the open borders of the European Union facilitate such crimes because of the ease of movement, this is not the case. Perhaps such crimes are more prevalent in more urbanized areas, as a study by Kinney et al. (2008) suggests. On the other hand, the problem could be viewed through local economic and social factors. For example, more roads and more cars are indicative of a region’s developing infrastructure and a wealthier society, and this can cause changes in crime (Allen, 1996; Kitchen, 2006). Moreover, an increase in the wealth of society may also bring about a greater focus on white collar crime such as fraud which is harder for police to detect.

We also observed an inverse relationship between registration and reregistration in pre-working age and in working age, and a lower crime rate. This is consistent with the results obtained in India where internal migration could not be confirmed to be responsible for an increase in crime (Debnath & Roy, 2013). One characteristic of the Opole province is the high migration of residents that is related to work or study. Residing in a particular place for a short period of time is not conducive to increased criminal activity as described by crime pattern theory. Potential criminals obtain information about achieving illegal goals from their legal daily life. It is accepted that most criminals behave normally on a daily basis and that criminals spend most of their time being involved in normal social activities rather than in committing crimes. Their familiarity with a city is thus achieved through their legal and everyday activities connected with locations like home, work, school, shopping areas, etc. (Branthingham & Branthingham, 1993).

Finally, as already dealt with in geographical criminology (Hu et al., 2018; Law et al., 2013), we also believe that the Bayesian approach is a useful tool for detecting patterns in crime statistics and, thus, is helpful in police work and should be used on a wider scale. In criminology of place the use of such methods is crucial because it helps create crime analyses that are predictable. The advantages of such solutions in police work are not in doubt as they help solve crime as noted by Weisburd et al. (2012).

## Conclusions

Based on the collected criminal data and the applied geostatistical methods the following general conclusions can be drawn from this study:

1. As already confirmed by other authors in the past Bayesian spatio-temporal methods can be successfully applied in criminological research. However, this requires efficient scientific and practical cooperation between statisticians and the police;
2. By overlapping Bayesian spatio-temporal models of the relative risk of crime and their increments, we obtained a geographical pattern of cold-spots and hot-spots with extremely different (reduced and increased) crime rates in the region. The effectiveness of this new mapping technique is confirmed by the compared indicators of crimes recorded in specific clusters;
3. On the basis of several dozen analyzed demographic and socio-economic-related population characteristics, for strictly scientific and cognitive purposes, by applying Bayesian modeling, we selected the most likely risk factors that may be a premise for committing crimes in the region in the five-year period studied;
4. The proposed analysis may become an additional geostatistical control instrument supporting the management and deployment of the local police and can be easily applied based on the available police crime records and public statistics;
5. The research technique offered requires scientific and practical verification in the assessment of its usefulness in police work. At the same time we trust that we are supplementing the missing statistical methodology in the Polish geography of crimes.

## Study limitations

The empirical results reported herein should be considered in light of some limitations. The first is the retrospective and non-experimental nature of the study (at this point, additionally, a lack of probability sampling of the data considered). But we have shown that even then, using easily available methods, a quick data analysis can be performed and practical results can be delivered. The second aspect concerns the overall presentation of the recorded crime rates in the region without a detailed analysis of the types of crime. The main intention of this study was, however, of an instructive nature both for academic and police personnel for better administrative management or police deployment and not a detailed search for specific cause-and-effect relationships in the gathered crime and regional data. The last limitation is a strong regional focus. We are aware that the obtained results may be specific for the studied region and therefore we approach their generalization with caution.

## Supplementary Information

**Additional file 1.** Population, demographic, socio-economic, and crime numbers in Opole Province, Poland, in the years 2015–2019 (Excel sheets)

**Additional file 2.** Bailey’s relative risk regression (WinBUGS model)

**Additional file 3.** Congdon’s growth rate regression (WinBUGS model)

**Additional file 4.** Bailey's ecological regression (WinBUGS model)

**Additional file 5.** Sample data (text format)

## Data Availability

All Excel and text datasets as well as WinBUGS codes used in the analysis are shared as part of this publication (Additional files 1, 2, 3, 4, 5).
